# Transapical Beating-Heart Septal Myectomy for Obstructive Hypertrophic Cardiomyopathy With Anomalous Papillary Muscle Insertion

**DOI:** 10.1093/icvts/ivaf195

**Published:** 2025-08-20

**Authors:** Yue Chen, Eduard Quintana, Jing Fang, Yani Liu, Xiang Wei

**Affiliations:** Division of Cardiovascular Surgery, Tongji Hospital, Tongji Medical College, Huazhong University of Science and Technology, Wuhan 430030, China; Key Laboratory of Organ Transplantation, Ministry of Education, NHC Key Laboratory of Organ Transplantation; Key Laboratory of Organ Transplantation, Chinese Academy of Medical Sciences, Wuhan, 430030, China; Department of Cardiovascular Surgery, Hospital Clinic de Barcelona, University of Barcelona, Barcelona, 08036, Spain; Division of Cardiovascular Surgery, Tongji Hospital, Tongji Medical College, Huazhong University of Science and Technology, Wuhan 430030, China; Key Laboratory of Organ Transplantation, Ministry of Education, NHC Key Laboratory of Organ Transplantation; Key Laboratory of Organ Transplantation, Chinese Academy of Medical Sciences, Wuhan, 430030, China; Department of Medical Ultrasound, Tongji Hospital, Tongji Medical College, Huazhong University of Science and Technology, Wuhan 430030, China; Division of Cardiovascular Surgery, Tongji Hospital, Tongji Medical College, Huazhong University of Science and Technology, Wuhan 430030, China; Key Laboratory of Organ Transplantation, Ministry of Education, NHC Key Laboratory of Organ Transplantation; Key Laboratory of Organ Transplantation, Chinese Academy of Medical Sciences, Wuhan, 430030, China

**Keywords:** obstructive hypertrophic cardiomyopathy, septal myectomy, anomalous papillary muscles, transapical beating-heart septal myectomy

## Abstract

**Objectives:**

Anomalous papillary muscles (PMs) directly inserted into the anterior mitral valve (MV) constitute an infrequent anomaly in obstructive hypertrophic cardiomyopathy (HCM). This study sought to evaluate the efficacy and safety of a novel approach with transapical beating-heart septal myectomy to relieve obstruction while avoiding abnormal PM manipulation.

**Methods:**

Among 439 patients with obstructive HCM from March 2023 to February 2024, 27 patients (6.2%) were diagnosed with anomalous PM directly inserted into the anterior mitral leaflet. Isolated myectomy without PMs intervention was performed in these patients with a transapical beating-heart septal myectomy approach.

**Results:**

The median age of patients was 54 (47-60) years. The abnormal PMs insertion into the body (type I/II) and only the free edge (type III) of anterior MV leaflet were 21 and 6 patients, respectively. There was no operative death, septal perforation, conversion to sternotomy, blood transfusion, and no patients with preoperative normal conduction required a pacemaker. The resting left ventricular outflow tract gradient decreased from 110 (70-121) mm Hg at baseline to 10 (8-21) mm Hg at 12 months. Reduction to a mitral regurgitation (MR) grade ≤ 1+ was achieved in 25 (92.6%) patients at 12 months. At follow-up, 25 (92.6%) patients returned to New York Heart Association (NYHA) class I.

**Conclusions:**

In selected patients with sufficient septal thickness and no intrinsic MV pathology, transapical beating-heart septal myectomy may provide obstruction and MR relief without direct PMs intervention. This new approach without sternotomy or cardiopulmonary bypass increases the options for this infrequent condition.

**Clinical registration number:**

NCT05332691

## Graphical abstract

**Figure:**
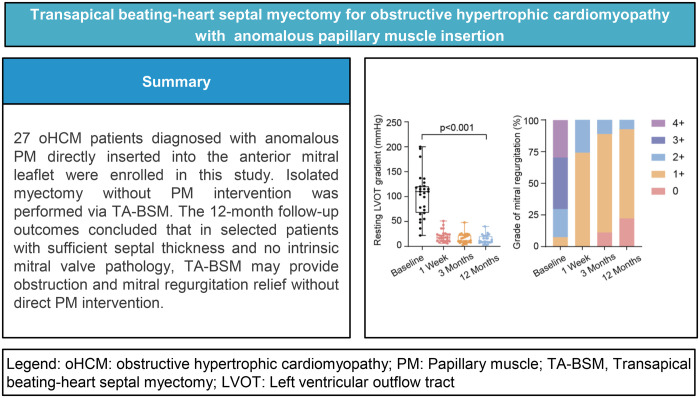

## INTRODUCTION

Hypertrophic cardiomyopathy (HCM) is the most common heritable heart disease.^1^ Approximately two-thirds of symptomatic patients present left ventricular outflow tract (LVOT) obstruction, defined as a left ventricular gradient over 30 mm Hg at rest or with provocation.^2^ Surgical septal myectomy remains the gold standard for obstruction relief in obstructive HCM (oHCM).^3–5^ However, septal myectomy is a difficult procedure that requires sternotomy, cardiopulmonary bypass (CPB), and cardioplegic arrest of the heart. The risks, although low, in these setting, may include complete heart block, ventricular septal defect, or injury to the aortic or mitral valves (MVs).^6^ LVOT obstruction is not uniquely due to septal hypertrophy, but a result from a complex interaction between the septal morphology, the mitral apparatus, papillary muscles (PMs), myocardial bands, and myocardial contractility. Among these, direct insertion of an anomalous PM into the anterior MV leaflet is one well-recognized phenotype that further contributes to obstruction. First, the location of the anomalous muscle in relation to the septum may predispose to abnormal contact below the level of the mitral leaflets, namely midventricular obstruction.^7^ In addition, the traction offered by anomalous PMs may trigger systolic anterior motion (SAM) of mitral leaflets, which beyond obstruction may result also in dynamic mitral regurgitation (MR).^7^ Finally, some abnormal PMs may also occupy space in the LVOT creating a conflict of space and fixed obstruction. During open transaortic SM, the excision of anomalous PMs has been proposed to abolish all forms of intraventricular obstruction, SAM, and secondary MR.^8^ Despite direct operative vision, full intraventricular control remains a challenge in some patients resulting in non-neglectable risk for reoperations, even in expert hands.^9^ Moreover, the contribution of anomalous PM resection on SAM and associated MR relief is hard to be evaluated under cardioplegic conditions.

We envisioned that patients with abnormal PM connections into MV could be treated by septal resection if enough space could be created in the LVOT.^10–12^ In this study, we describe a new, prospectively collected cohort, distinct from that previously published,^12^ and focus on patients with anomalous PMs that underwent an isolated septal myectomy approach.

## METHODS

### Study patients

From March 2023 to February 2024, oHCM patients with unresponsive symptoms despite optimal medical therapy or intolerant to drugs underwent septal myectomy. The study was conducted in accordance with the International Council for Harmonization and the Declaration of Helsinki. The protocol was approved by the Ethics Committee of the Tongji Medical College (approval Nos.: 2022-S013, 2022-S013-1 to 2022-S013-6), and the study was registered at ClinicalTrials.gov (NCT05332691). Any collection and storage of data from research participants for multiple and indefinite use were consistent with requirements outlined in the WMA Declaration of Taipei. The ethics committee approved the establishment and monitor ongoing use of databases. Before enrollment, all patients underwent a comprehensive clinical and multimodality imaging evaluation, including transthoracic echocardiography (TTE), transoesophageal echocardiography (TEE) when necessary, and cardiac magnetic resonance imaging (CMR). Patients were eligible according to following criteria: (1) at least 8 years old, (2) resting or provoked LVOT gradient over 50 mm Hg, and (3) maximal septal thickness over 15 mm. The key exclusion criteria were (1) severe cerebrovascular disease, (2) left ventricular systolic dysfunction (LV ejection fraction < 40%), (3) pregnancy, (4) poor surgical candidacy (Child-Pugh class C and other general conditions precluding open heart surgery), and (5) other structural heart valve abnormalities with indication for surgery.

Before enrollment, all patients provided written informed consent to participate in the study.

### Diagnosis

The evaluation of the cardiac structure and function was provided in the supplemental materials, as described previously.^10^ According to the attachment site on the ventricular side of the MV leaflet, the anomalous PM is divided into 3 subtypes: insertion of PM bundles directly into the base of the ventricular side of the anterior MV leaflet (type I anomaly), insertion into the body and free edge of the leaflet (type II anomaly), and insertion involving only the free edge of the leaflet (type III anomaly). Due to the limitation of imaging techniques, type I and type II PM insertion are not able to be determined without direct vision, and thus were discussed together in this review.

### Beating-heart myectomy device

The beating-heart myectomy device (BMD, Vass M1, Vass Medical Technology Corporation, Ltd) was developed as previously outlined by our team.^10^ The device comprises a paraxial puncture needle, resection blade, resection tube, needle slider, and resection blade handle. The resection tube features a bullet-shaped head, resection window, suction tube, and polyurethane coating. A resection window is located on the sidewall of the head portion of the sleeve tube. To prevent air bubbles from entering the bloodstream, normal saline is flushed into the resection tube of the BMD to eliminate air from the device’s internals. Under the guidance of TEE, the device is inserted into the left ventricle with its resection blade enclosed and is positioned at the hypertrophic myocardium. The resection window is opened via the puncture needle and the tubular blade pulled back and is then aligned to the target myocardium. The paraxial puncture needle secures the targeted tissue by piercing it, followed by the upward advancement of the tubular blade to the device’s tip for tissue resection. Finally, the device is withdrawn from the ventricle, thereby completing one resection. Registered clinical trials for the device (ChiCTR2400087414) are currently underway in China.

### TA-BSM procedure

**Figure 1.**
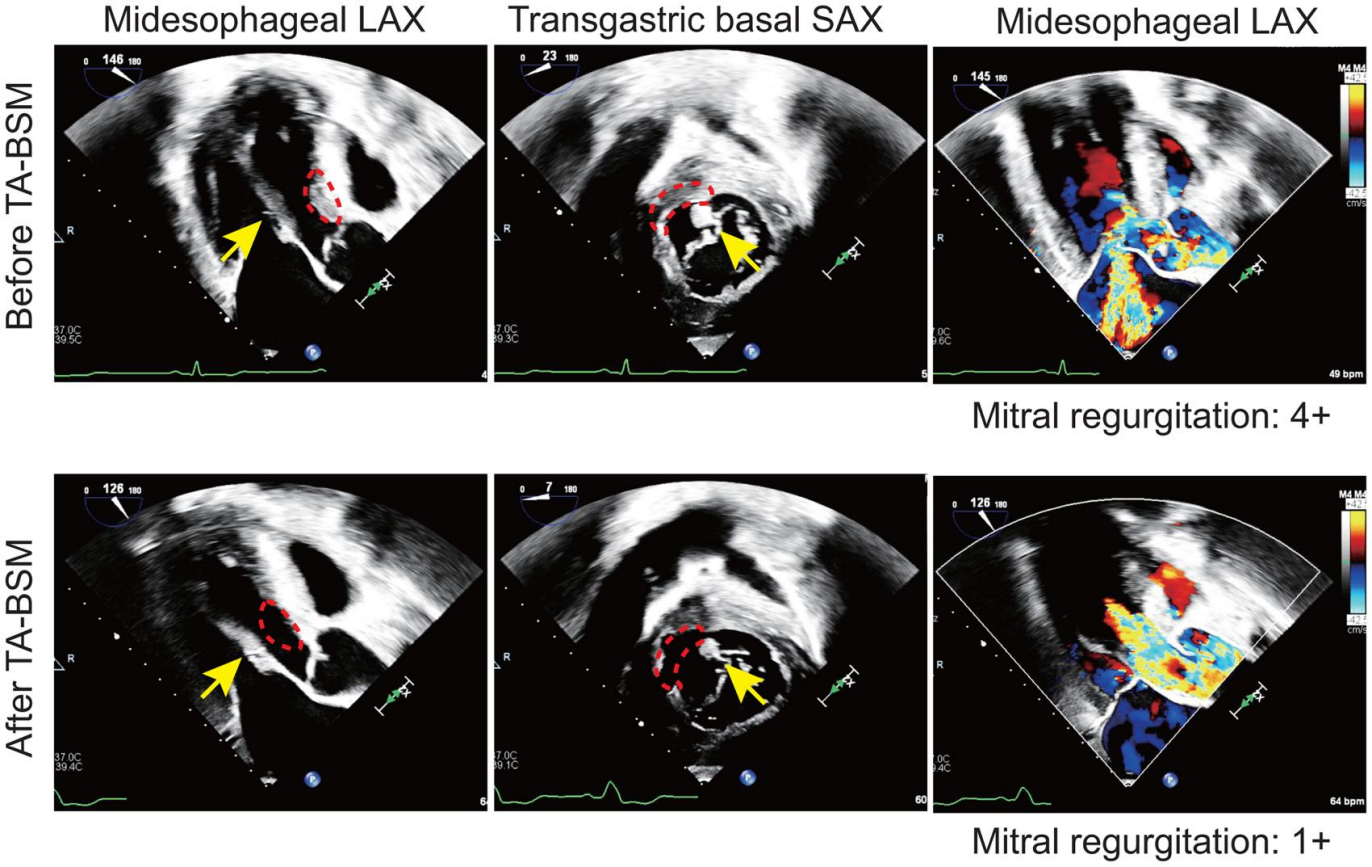
Representative Transoesophageal Echocardiographic Image During Transapical Beating-Heart Septal Myectomy. The echocardiographic images showing the long-axis (LAX) and short-axis (SAX) views of transoesophageal echocardiography before (top) and after (bottom) TA-BSM. The resected zones are marked by the red dotted curve and the yellow arrows identify anomalous insertion of PMs into the mitral apparatus

A left anterolateral incision was performed in the fifth or sixth intercostal space to expose left ventricular apex. Double circumferential purse-string sutures with Teflon pledgets were performed on the avascular area of left ventricular apex. After full heparinization (4 mg/kg), the apex was punctured and a guidewire navigated towards the aortic root. Invasive intraventricular pressure was measured. Then, the dilator was inserted into the left ventricle to facilitate a smooth BMD entrance. The device was introduced gently in contact to the septum and advanced towards the LVOT. Position was verified and rechecked with TEE, the targeted myocardium was captured with advancement of the needle and a basal septal myectomy with the rotatory blade performed. The procedure was performed on the beating heart without CPB support.^10^ Multiple resections were performed under TEE navigation to ensure the necessary gain in LVOT space to eliminate obstruction and SAM. Invasive intraventricular and echocardiographic measurements at rest and post induced premature ventricular contraction were obtained (**Figure 1** and **Figure S1**).

For the patients with anomalous PMs, the recognition of PMs-mediated SAM is key to determine the zone that most contributes to LVOT/midventricular obstruction. Before resection, the encroachment zone of MV to septal was described with TEE and the precise zone to be targeted was delineated. This is carried with the intention to create enough LVOT space to avoid both dynamic and fixed obstruction caused by PM space occupation. Notably, the strategy with TA-BSM avoided any attempt to resect abnormal PMs that could induce further MR (**Video 1**).

### Statistics

Quantitative data are expressed as median (interquartile range [IQR]). The normality of quantitative variables was tested by Shapiro-Wilk test. Comparisons of continuous variables between baseline and 12 months postoperatively were conducted using the paired sample t-test for normally distributed data and the Wilcoxon matched-pairs signed rank test for non-normally distributed data. Categorical variables are described by frequencies and percentages. For categorical variables, the McNemar’s test for binary variables and the Wilcoxon matched-pairs signed rank test for ordinal variables were performed to compare the data between baseline and postoperative 12 months. Statistical analyses were performed with SPSS software version 23.0 (IBM). Statistical significance was presented with a 2-sided *P* value less than 0.05.

## RESULTS

### Baseline characteristics

Among the 439 patients with oHCM, the abnormalities of the PM were described in **Table 1**. PM hypertrophy was the most common abnormality, observed in 282 (64.2%) patients in the entire cohort. Twenty-two males and 5 females were described as having direct insertion of the anomalous PM to the MV, and the median (IQR) age was 54 (47-60) years (**Table 2**). The anomalous PM inserted into the body of the anterior MV leaflet (type I/II) and the free edge of the anterior leaflet (type III) were 21 (77.8%) and 6 (22.2%) patients, respectively. Among the enrolled patients, chest pain and dyspnoea were present in 16 (59.3%) and 21 (77.8%) patients, respectively. One patient had been diagnosed with Fabry disease and underwent kidney transplantation 5 years before. Two patients with prior percutaneous radiofrequency had residual obstruction. All patients were on medicine therapy, including beta-blockers and calcium antagonist preoperatively, and SAM of MV was identified on TTE in 23 (85.2%). Among the 27 patients, 3 (11.1%) showed latent LVOT obstruction with a resting LVOT gradient <50 mm Hg. The baseline LVOT gradient (LVOTG) was 110 (70-121) mm Hg at rest, ranging from 22 to 200 mm Hg. Subaortic and long-segment hypertrophy were detected in 16 (59.3%) and 11 (40.7%) patients, respectively.

**Table 1.** Structural and Functional Abnormalities of PMs

Parameters	All patients
PM hypertrophy	282 (64.2%)
Direct PM insertion into anterior mitral leaflet (anomalous PM)	27 (6.2%)
Doubly bifurcated PMs	57 (13.0%)
Accessory PMs	117 (26.7%)
Apical PM displacement	95 (21.6%)

Abbreviation: PM: papillary muscle.

**Table 2.** Baseline Clinical Characteristics of Patients with Direct PM Insertion

Parameters	All patients (*n* = 27)
Age (years)	54 (47-60)
Female	5 (18.5%)
Latent LVOTO	3 (11.1%)
Body surface area (m^2^)	1.82 (1.73-1.92)
Subtypes of HCM	
Subaortic	16 (59.3%)
Long segment	11 (40.7%)
Subtypes of anomalous PM	
Type I/II	21 (77.8%)
Type III	6 (22.2%)
Resting LVOTG (mm Hg)	110 (70-121)
<50	3 (11.1%)
50-100	8 (29.6%)
>100	16 (59.3%)
Symptoms	
Chest pain	16 (59.3%)
Dyspnoea	21 (77.8%)
Amaurosis	4 (14.8%)
Syncope	3 (11.1%)
Postprandial aggravation of symptom	3 (11.1%)
Palpitation	8 (29.6%)
Sudden cardiac death index (%)	3.20 (2.31-3.54)
EuroSCORE II (%)	0.69 (0.62-0.88)

Data are median (IQR), or *n* (%).

Abbreviations: EuroSCORE II: European System for Cardiac Operative Risk Evaluation II; LVOTG: left ventricular outflow tract gradient; LVOTO: left ventricular outflow tract obstruction.

### Perioperative details

Among the 27 patients, the median weight of resected myocardium was 4.4 (3.4-5.7) g and the median number of resections was 5 (4-8) (**Table 3**). No patients were converted to median sternotomy or required CPB. There was no need for blood transfusion in the perioperative period. There was no in-hospital or 30-day mortality in the study population. New-onset left bundle branch block occurred in 18 (66.7%) patients, and 2 patients with preoperative complete right bundle branch block underwent permanent pacemaker implantation due to high-grade atrioventricular block after TA-BSM. There was no direct iatrogenic atrioventricular node damage and stroke as none of the patients with preoperative normal intraventricular required a pacemaker. The median operation time and ventilation time after the operation was 1.1 (0.7-1.3) h and 4.1 (3.4-5.6) h. The median intensive care unit length of stay was 25.4 (21.2-36.5) h.

**Table 3.** TA-BSM Procedure-Associated Perioperative Parameters and Clinical Events

Parameters	All patients (*n* = 27)
Perioperative data	
Weight of resected myocardium (g)	4.4 (3.4-5.7)
Number of resections	5 (4-8)
Operation time (h)	1.1 (0.7-1.3)
Duration of ventilation (h)	4.1 (3.4-5.6)
ICU stay (h)	25.4 (21.2-36.5)
Red blood cell transfusion	0 (0.0%)
New-onset left bundle branch block	18 (66.7%)
Permanent pacemaker implantation^a^	2 (7.4%)
Major adverse events	
Iatrogenic ventricular septal perforation	0 (0.0%)
Left ventricular apical tear	0 (0.0%)
Median sternotomy conversion	0 (0.0%)
Iatrogenic valvular injury	0 (0.0%)
Iatrogenic atrioventricular blockage	0 (0.0%)
Stroke	0 (0.0%)
In-hospital mortality	0 (0.0%)

Data are median (IQR) or *n* (%).

a In 2 patients that had preoperative right bundle branch blockage.

No major adverse events happened among the 27 patients. One patient with high resting LVOTG (107 mm Hg) and severe MR (4+) had significant obstruction relief (resting LVOTG 15 mm Hg) after resection. As expected, despite SAM resolution, MR did not regress fully due to the presence of chronic concomitant organic mitral derangement, and we elected to apply a transcatheter mitral clamp through the same apical port. The result was a 2+ MR that is being followed closely.

### Postoperative outcomes

**Figure 2.**
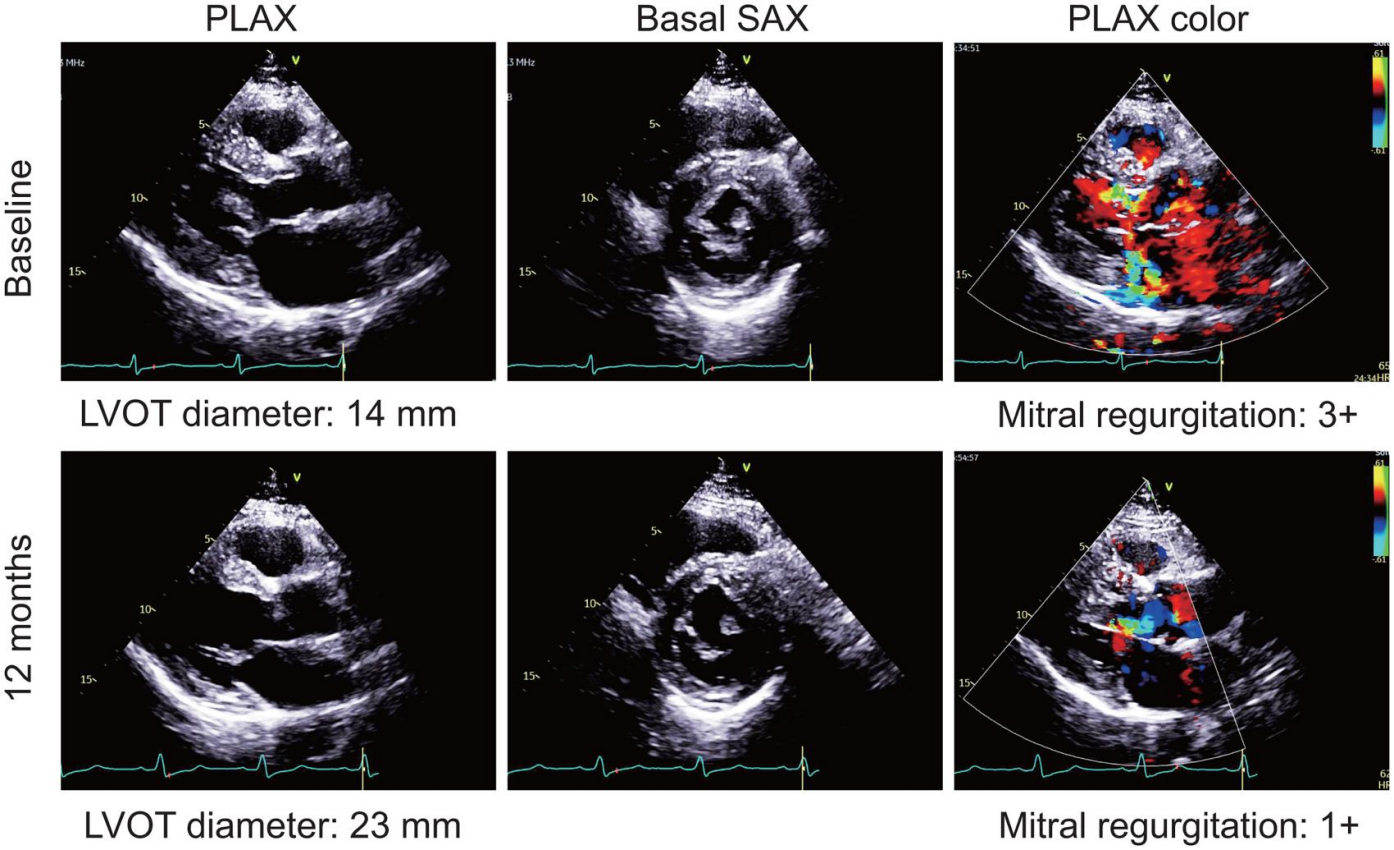
Representative Transthoracic Echocardiographic Images After Transapical Beating-Heart Septal Myectomy. The echocardiographic images showing the morphology and MR at baseline (top) and 12 months (bottom) after TA-BSM. Abbreviations: PLAX: parasternal long-axis view; SAX: short-axis view

**Figure 3.**
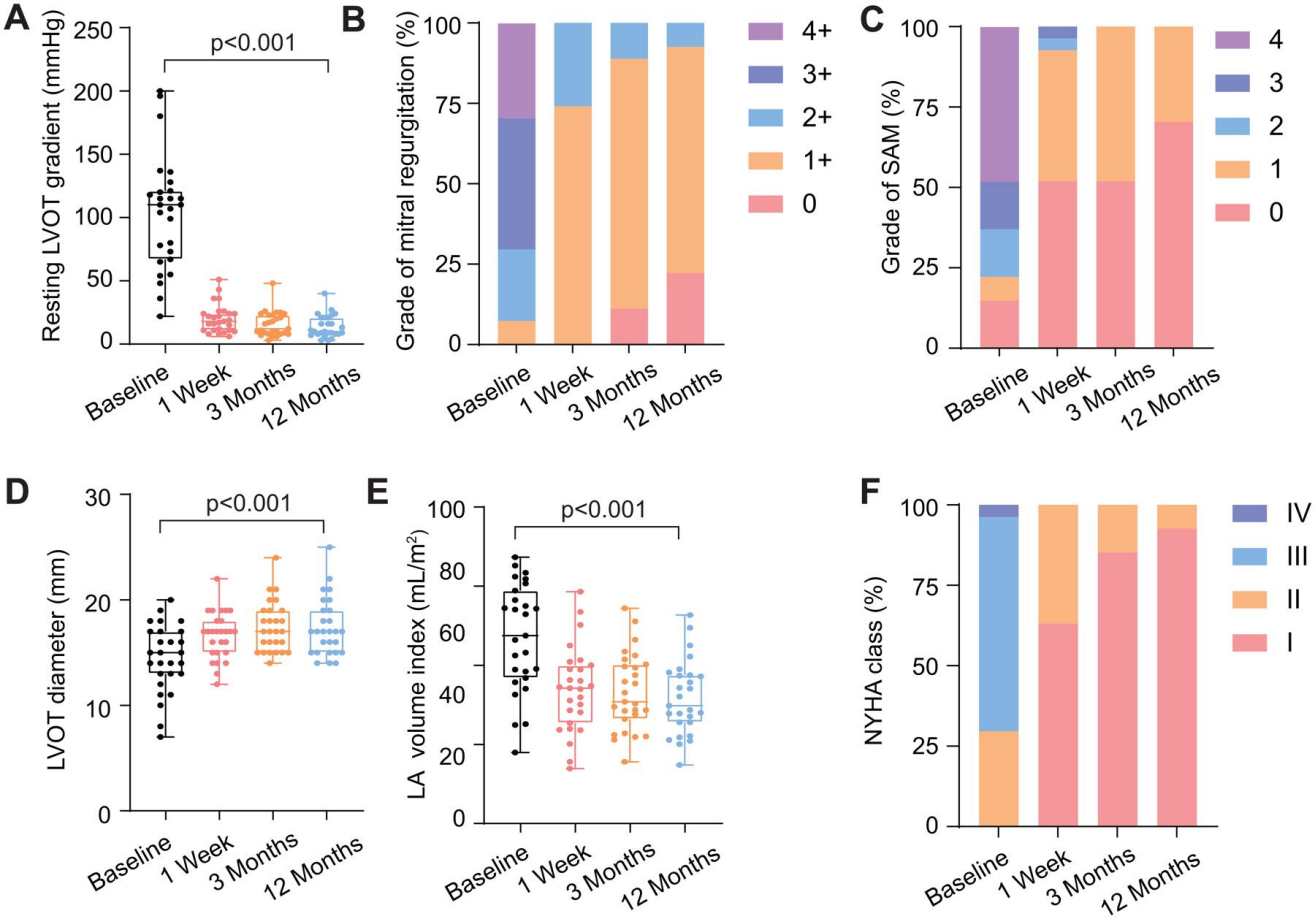
Clinical Parameters and Follow-up Outcomes after Transapical Beating-Heart Septal Myectomy. (A) Resting left ventricular outflow tract gradient (LVOTG) as indicated. (B) Grade of mitral regurgitation as indicated. (C) Grade of systolic anterior motion (SAM) as indicated. (D) Left ventricular outflow tract (LVOT) diameter as indicated. (E) Left atria (LA) volume index as indicated. (F) New York Heart Association (NYHA) class as indicated

Follow-up was complete for all patients at 3 and 12 months, and the post-procedure characteristics are shown in **Table 4**. The representative morphologic alternations of TTE of 1 patient are shown in **Figure 2** and **Figure S2**. Among the 27 patients, the median resting LVOTG reduced from 110 (70-121) mm Hg at baseline to 10 (8-21) mm Hg at 12 months (*P* < 0.001) (**Figure 3A**). A maximal LVOTG < 50 mm Hg was reached in 25 (92.6%) patients. Reduction to a MR grade ≤ 1+ was achieved in 25 (92.6%) patients (**Figure 3B**). Mitral valve SAM was significantly improved during follow-up and no patients had more than moderate SAM (≥grade 2+) at 12 months (**Figure 3C**). The LVOT diameter increased at 12 months (median [IQR], 17 [15-19] mm) versus baseline (median [IQR], 15 [13-17] mm) (*P* < 0.001) (**Figure 3D**). Each ventricular septum region decreased in thickness, and the average maximal thickness was less than 15 mm at 12 months. Moreover, LV diastolic function reflected by left atrial (LA) volume index and E/e’ was significantly improved at 12 months, accompanied by enlarged left ventricular end-diastolic volume (LVEDV) index (**Figure 3E**). The New York Heart Association (NYHA) functional class improved significantly and 25 (92.6%) patients were in NYHA class I at 12-month follow-up (**Figure 3F**). Similarly, exercise capacity and quality of life in the enrolled patients were all improved at 12 months.

**Table 4.** Clinical Parameters and Follow-up Outcomes

	Baseline	1 week	3 months	12 months	*P* value^a^
Echocardiography	*N* = 27	*N* = 27	*N* = 27	*N* = 27	
Resting LVOTG (mm Hg)	110 (70-121)	18 (11-24)	12 (9-23)	10 (8-21)	<0.001
<30 mm Hg	1 (3.7%)	23 (85.2%)	26 (96.3%)	26 (96.3%)	
≥30 mm Hg	26 (96.3%)	4 (14.8%)	1 (3.7%)	1 (3.7%)	
Maximal LVOTG (mm Hg)	110 (73-121)	NA	18 (12-27)	15 (11-25)	<0.001
<50 mm Hg	0 (0.0%)	NA	25 (92.6%)	25 (92.6%)	
≥50 mm Hg	27 (100%)	NA	2 (7.4%)	2 (7.4%)	
Mitral regurgitation					<0.001
0	0 (0.0%)	0 (0.0%)	3 (11.1%)	6 (22.2%)	
1+	2 (7.4%)	20 (74.1%)	21 (77.8%)	19 (70.4%)	
2+	6 (22.2%)	7 (25.9%)	3 (11.1%)	2 (7.4%)	
3+	11 (40.7%)	0 (0.0%)	0 0.0%)	0 0.0%)	
4+	8 (29.6%)	0 (0.0%)	0 (0.0%)	0 (0.0%)	
SAM					<0.001
0	4 (14.8%)	14 (51.9%)	14 (51.9%)	19 (70.4%)	
Grade 1	2 (7.4%)	11 (40.7%)	13 (48.1%)	8 (29.6%)	
Grade 2	4 (14.8%)	1 (3.7%)	0 (0.0%)	0 (0.0%)	
Grade 3	4 (14.8%)	1 (3.7%)	0 (0.0%)	0 (0.0%)	
Grade 4	13 (48.1%)	0 (0.0%)	0 (0.0%)	0 (0.0%)	
LVOT diameter (mm)	15 (13-17)	17 (15-18)	17 (15-19)	17 (15-19)	<0.001
Basal anterior IVSd (mm)	19 (18-22)	13 (12-15)	12 (11-14)	11 (10-14)	<0.001
Basal posterior IVSd (mm)	17 (14-20)	12 (11-14)	12 (10-13)	11 (9-13)	<0.001
Mid anterior IVSd (mm)	17 (15-20)	14 (12-15)	13 (11-15)	12 (11-14)	<0.001
Mid posterior IVSd (mm)	18 (17-20)	13 (12-16)	12 (11-14)	11 (10-14)	<0.001
LVEDV index (mL/m^2^)	56.0 (42.5-63.9)	49.8 (42.0-58.2)	58.9 (49.4-65.9)	60.1 (51.5-67.2)	<0.001
LA volume index (mL/m^2^)	47.5 (37.6-57.7)	34.2 (26.8-39.5)	30.8 (27.0-40.0)	29.8 (25.6-37.5)	<0.001
* E*/*e*’					<0.001
<15	10 (37.0%)	15 (55.6%)	16 (59.3%)	19 (70.4%)	
≥15	17 (63.0%)	12 (44.4%)	11 (40.7%)	8 (29.6%)	
NT-proBNP (pg/mL)	1181 (696-2270)	1334 (657-2054)	498 (248-954)	437 (185-843)	<0.001
Quality of life					
NYHA class					<0.001
NYHA class I	0 (0.0%)	17 (63.0%)	23 (85.2%)	25 (92.6%)	
NYHA class II	8 (29.6%)	10 (37.0%)	4 (14.8%)	2 (7.4%)	
NYHA class III	18 (66.7%)	0 (0.0%)	0 (0.0%)	0 (0.0%)	
NYHA class IV	1 (3.7%)	0 (0.0%)	0 (0.0%)	0 (0.0%)	
6-MWT (m)	358 (329-404)	NA	466 (403-497)	486 (434-519)	<0.001
KCCQ score	64 (52-73)	NA	86 (80-91)	88 (82-92)	<0.001

Data are median (IQR), or *n* (%).

Abbreviations: *E*/*e*’: the ratio between early mitral inflow velocity and mitral annular early diastolic velocity; IVSd: interventricular septal thickness in diastole; KCCQ: Kansas City Cardiomyopathy Questionnaire; LA: left atria; LVEDV: left ventricular end-diastolic volume; LVOT: left ventricular outflow tract; LVOTG: left ventricular outflow tract gradient; NT-proBNP: N-terminal pro-brain natriuretic peptide; NA: not applicable; NYHA: New York Heart Association; SAM: systolic anterior motion; 6-MWT: 6-minute walking test.

a 12 months versus baseline.

## DISCUSSION

This study presents a novel operative approach with TA-BSM for oHCM in the presence of abnormal PM connecting into the MV. The new strategy reformulates classical management through a PM-sparing approach consisting of isolated tailored septal myectomy. Observational data and historical recommendations have suggested that open surgical myectomy and abnormal PM excision is required to achieve satisfactory outcomes.^8^^,^^13^^,^^14^ In fact, the presence of this type of abnormality qualifies patients for open heart surgery instead of alcohol septal ablation, according to clinical practice guidelines.^15^^,^^16^ In this cohort, sufficient septal myectomy without PM intervention was performed and the outcome was encouraging. The 12-month follow-up showed that there was general relief of resting and provocable LVOT obstruction, along with significant reduction of MR in these patients. The avoidance of excision of aberrantly inserted PMs through TA-BSM challenges the traditional approach for anomalous PMs management and simplifies the treatment for these complicated oHCM phenotypes, potentially expanding the adoption of TA-BSM. While the PMs were not resected, we cannot exclude indirect mechanical effects or functional changes resulting from septal reshaping.

Anomalous and direct PM insertion into anterior mitral leaflet is an uncommon but a nonnegligible mitral abnormality in HCM and has been thought to contribute to recurrent LVOT obstruction after septal myectomy.^8^ In general, there is agreement that resection of PM inserting into the body of the anterior MV leaflet (type I and II) contributes to obstruction relief.^8^ Type III anomalous PM may have no contribution to subaortic obstruction, and resection might only be necessary in the presence of midventricular obstruction. Occasionally, even after resolution of obstruction through appropriate open classic septal myectomy and concomitant PM resection, residual MR persist, despite absence of SAM or flail. Whether these situations are due to induced deformation of the MV, after dividing the PM, is unclear. Literature suggests that the MV, at times, may need to be replaced within the frame of treatment for this condition.^8^ This could be another argument to attempt a PM-sparing approach as it avoids unpredictable functional deformation of the MV once it is released from the abnormal attachment. From an intuitive standpoint, if enough intracavitary space can be generated to abolish SAM and midventricular obstruction, then a simplified approach with TA-BSM could be indicated. This seems defensible provided that no fixed LVOT obstruction is left behind because of LV space stolen by the PM.

The data reported in here support this approach in selected oHCM patients. Patients might be candidates for this novel approach when sufficient septal tissue (eventually >17 mm) is available at the targetable region. The advantage is that with TA-BSM, a more precise resection can be achieved with real-time echocardiographic support minimizing the need to debulk further the cavity or manipulating the PM. This approach offers a fresh perspective for complicated oHCM and challenges established recommendations based on previous observational data. Careful assessment of the septal morphology, valvular and subvalvular apparatus is necessary for operative planning and define candidacy for this PM-sparing approach. As recommended, TTE is the initial diagnostic modality, given its noninvasive nature.^15^^,^^16^ Comprehensive evaluation of HCM, with preoperative CMR and intraoperative TEE, are routinely performed to further evaluate all SM patients before skin incision.^17^ Anomalous PMs are often well-identified, including site of insertion, by 3-dimensional TEE.^7^ Due to superior spatial resolution and multiplanar capabilities, CMR has advantages in further depicting abnormal morphology and location of PMs.^18^ Alternatively, computerized tomographic angiography may obtain equivalent-to-CMR morphologic evaluation while providing information on coronary abnormalities. These multimodality imaging may provide information on the contribution of PM to obstruction, SAM, and MR in order to determine whether the sufficient septectomy will correct the abnormal pathophysiology.

SAM remains a concerning phenomenon in oHCM and is associated with LVOT obstruction and MR in almost 70% of HCM patients.^19^ Previous studies indicate that beyond septal hypertrophy, SAM is also a consequence of mitral apparatus structural anomalies, geometric factors, hyperdynamic LV, and its pathophysiological presence is mainly justified by abnormal flow vortices (dragging forces). It is understandable that anomalous PMs may bring the MV anteriorly into the LVOT predisposing to SAM^7^ but the extend of this phenomenon remains difficult to quantify. Previous studies conclude that successful surgical approach to these anomalous PM requires a sufficient excision from its attachment on the body of the anterior leaflet to the level of the origin of PM at the time of conventional septal myectomy, aiming to relieve obstruction and SAM.^20^ Due to the nature of the classic operation being carried under cardioplegic arrest conditions, it is impossible to ensure in real-time a full relief of obstruction and MR. In this line, whether myectomy and PM resection will lead to MR resolution is hard to predict in the arrested heart, risking patients to abandon CPB with residual (due to SAM) or induced MR. In TA-BSM, intraoperative real-time TEE monitoring determines the dominant location of SAM and facilitates a directed and precise resection. Importantly, the BMD can be located anywhere in the septum, leading to improved identification of the desired myocardium. As a result, TA-BSM may prevent unnecessary myocardial resection or excessive valve treatments, which occasionally could lead to the need of direct mitral intervention (including valve replacement).

The drawback of TA-BSM is that in the event that obstructive physiology/MR persisted, there are limited options to deal with this through the same access. Conversion to sternotomy or right thoracotomy may be necessary to fully correct abnormal residual derangements. Nevertheless, in our opinion, the presented surgical option is a new tool that may help widen the choices for oHCM.

## CONCLUSIONS

In selected patients with sufficient septal thickness and no intrinsic MV pathology, TA-BSM may provide obstruction and MR relief without direct PM intervention. This new approach without sternotomy or CPB increases the options for this infrequent condition.

## Supplementary Material

ivaf195_Supplementary_Data

## Data Availability

The raw data for this study can be obtained from the corresponding author upon a reasonable request.

## References

[1] Maron BJ, Desai MY, Nishimura RA, et al Diagnosis and evaluation of hypertrophic cardiomyopathy: JACC state-of-the-art review. J Am Coll Cardiol. 2022;79:372-389. 35086660 10.1016/j.jacc.2021.12.002

[2] Veselka J, Anavekar NS, Charron P, et al Hypertrophic obstructive cardiomyopathy. Lancet. 2017;389:1253-1267. 27912983 10.1016/S0140-6736(16)31321-6

[3] Maron BJ, Desai MY, Nishimura RA, et al Management of hypertrophic cardiomyopathy. J Am Coll Cardiol. 2022;79:390-414. 35086661 10.1016/j.jacc.2021.11.021

[4] de Villarreal-Soto JE, Oteo-Domínguez JF, Martínez-López D, et al Extended septal myectomy versus alcohol septal ablation: clinical results at a national referral centre. Interdiscip Cardiovasc Thorac Surg. 2024;38:ivae058. 10.1093/icvts/ivae058 PMC11090985 38569884

[5] Rudenko KV, Lazoryshynets VV, Nevmerzhytska LO, et al Septal myectomy with mitral valve surgery in patients after alcohol septal ablation. Interact CardioVasc Thorac Surg. 2022;34:723-730. 35106584 10.1093/icvts/ivac010 PMC9070461

[6] Ward AF, Schaff HV, Dearani JA, et al Septal myectomy: how I teach it. Ann Thorac Surg. 2023;115:20-24. 36089072 10.1016/j.athoracsur.2022.08.041

[7] Nampiaparampil RG, Swistel DG, Schlame M, et al Intraoperative two- and three-dimensional transesophageal echocardiography in combined myectomy-mitral operations for hypertrophic cardiomyopathy. J Am Soc Echocardiogr. 2018;31:275-288. 29502589 10.1016/j.echo.2017.11.016

[8] Lentz Carvalho J, Schaff HV, Morris CS, et al Anomalous papillary muscles-implications in the surgical treatment of hypertrophic obstructive cardiomyopathy. J Thorac Cardiovasc Surg. 2022;163:83-89.e1. 32414597 10.1016/j.jtcvs.2020.04.007

[9] Minakata K, Dearani JA, Nishimura RA, et al Extended septal myectomy for hypertrophic obstructive cardiomyopathy with anomalous mitral papillary muscles or chordae. J Thorac Cardiovasc Surg. 2004;127:481-489. 14762358 10.1016/j.jtcvs.2003.09.040

[10] Fang J, Liu Y, Zhu Y, et al First-in-human transapical beating-heart septal myectomy in patients with hypertrophic obstructive cardiomyopathy. J Am Coll Cardiol. 2023;82:575-586. 37558369 10.1016/j.jacc.2023.05.052

[11] Fang J, Wang R, Liu H, et al Transapical septal myectomy in the beating heart via a minimally invasive approach: a feasibility study in swine. Interact CardioVasc Thorac Surg. 2019;30:303-311. 10.1093/icvts/ivz249 31642911

[12] Li J, Wei X. Transapical beating-heart septal myectomy for hypertrophic cardiomyopathy with latent obstruction. Eur J Cardiothorac Surg. 2024;65:ezad425. 10.1093/ejcts/ezad425 PMC10903174 38113423

[13] Nomura T, Harada Y, Suzaki Y, et al Left ventricular outflow tract obstruction due to anomalous insertion of papillary muscle. Circ J. 2004;68:1219-1222. 15564711 10.1253/circj.68.1219

[14] Mutsuga M, Tokuda Y, Fujimoto K, et al Surgery for anomalous papillary muscle directly into the anterior mitral leaflet. Ann Thorac Surg. 2021;111:1512-1518. 32980328 10.1016/j.athoracsur.2020.07.031

[15] Ommen SR, Mital S, Burke MA, et al 2020 AHA/ACC guideline for the diagnosis and treatment of patients with hypertrophic cardiomyopathy: executive summary: a report of the American College of Cardiology/American Heart Association Joint Committee on Clinical Practice Guidelines. Circulation. 2020;142:e533-e557. 33215938 10.1161/CIR.0000000000000938

[16] Authors/Task Force members, Elliott PM, Anastasakis A, et al 2014 ESC guidelines on diagnosis and management of hypertrophic cardiomyopathy: the task force for the diagnosis and management of hypertrophic cardiomyopathy of the European Society of Cardiology (ESC). Eur Heart J. 2014;35:2733-2779. 25173338 10.1093/eurheartj/ehu284

[17] Nagueh SF, Phelan D, Abraham T, et al Recommendations for multimodality cardiovascular imaging of patients with hypertrophic cardiomyopathy: an update from the American Society of Echocardiography, in collaboration with the American Society of Nuclear Cardiology, the Society for Cardiovascular Magnetic Resonance, and the Society of Cardiovascular Computed Tomography. J Am Soc Echocardiogr. 2022;35:533-569. 35659037 10.1016/j.echo.2022.03.012

[18] Rajiah P, Fulton NL, Bolen M, et al Magnetic resonance imaging of the papillary muscles of the left ventricle: normal anatomy, variants, and abnormalities. Insights Imaging. 2019;10:83. 31428880 10.1186/s13244-019-0761-3 PMC6702502

[19] Silbiger JJ. Abnormalities of the mitral apparatus in hypertrophic cardiomyopathy: echocardiographic, pathophysiologic, and surgical insights. J Am Soc Echocardiogr. 2016;29:622-639. 27146120 10.1016/j.echo.2016.03.003

[20] Rowin EJ, Maron BJ, Lesser JR, et al Papillary muscle insertion directly into the anterior mitral leaflet in hypertrophic cardiomyopathy, its identification and cause of outflow obstruction by cardiac magnetic resonance imaging, and its surgical management. Am J Cardiol. 2013;111:1677-1679. 23499271 10.1016/j.amjcard.2013.01.340

