# Supplementary material for: Transapical Beating-Heart Septal Myectomy for Obstructive Hypertrophic Cardiomyopathy With Anomalous Papillary Muscle Insertion
Source: Interdiscip Cardiovasc Thorac Surg. 2025 Aug 20;40(9):ivaf195. doi: 10.1093/icvts/ivaf195 (PMC12548028; doi:10.1093/icvts/ivaf195)
Supplement: ivaf195_Supplementary_Data [file ivaf195_Supplementary_Data.zip › Supplementary Material-ICVTS.docx]

**Supplementary materials**

**Echocardiography**

TTE was performed in all patients to evaluate the cardiac structure and function at baseline, followed by at 1 week, 3 and 12 months after operation. LVOT gradients were estimated using the modified Bernoulli equation based on the peak velocity, measured by continuous-wave Doppler echocardiography. Provocation tests were performed on the patients with a resting LVOT gradient < 50 mmHg via treadmill, repetitive squat-to-stand maneuver, or the Valsalva maneuver. The degree of MR was assessed by means of color flow Doppler, and divided into five levels: 0 (none), 1+ (mild), 2+ (moderate), 3+ (moderate to severe), and 4+ (severe). SAM of the mitral valve was graded as 0 (none), 1 (leaflet-septal distance > 10 mm), 2 (leaflet-septal distance ≤ 10 mm but no leaflet-septal contact), 3 (leaflet-septal contact < 30% of the systolic duration), 4 (leaflet-septal contact ≥ 30% of the systolic duration). Echocontrast was used if needed.

A comprehensive evaluation was performed to identify PM abnormalities in all patients. In this study, PM hypertrophy, direct PM insertion into anterior mitral leaflet, accessory PMs, apical PM displacement, doubly bifurcated PMs were distinguished via TTE and a single patient might present more than one abnormality. PM hypertrophy is defined as end-diastolic PM width over than 1.1 cm in the short-axis view. Accessory PMs is characterized by a third or fourth PM that separates from the anterolateral and posteromedial PMs. Apical PM displacement means the shift of the PM origin towards the apex. Doubly bifurcated PMs is defined as the bifurcation of anterolateral or posteromedial PMs, and the bifurcated PMs are both connected to the MV leaflets via the tendon cords.

**
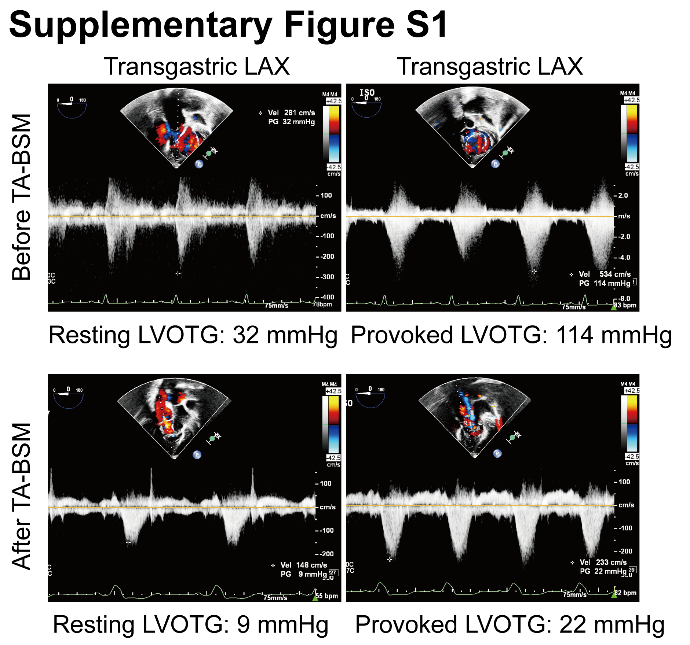
**

Supplementary Figure S1, related to Figure 1. Representative transesophageal echocardiographic image during transapical beating-heart septal myectomy. The echocardiographic images showing the resting and provoked LVOTG before (top) and after (bottom) TA-BSM. LAX, long-axis.


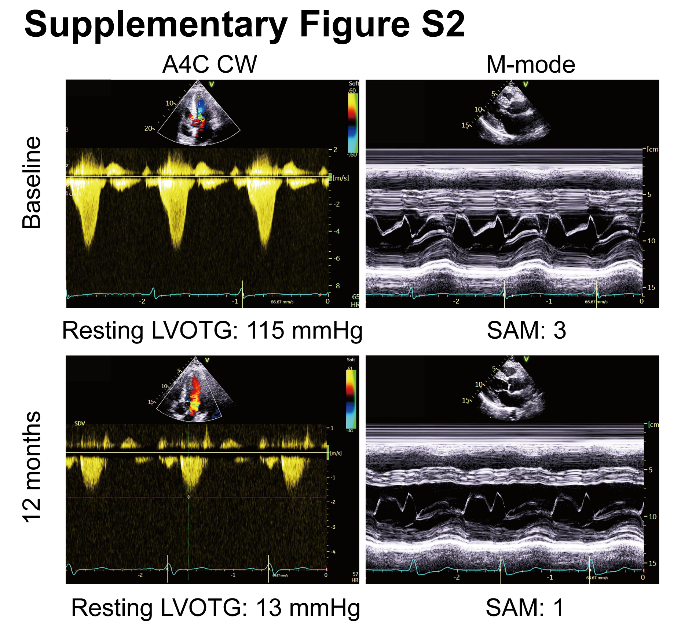


Supplementary Figure S2, related to Figure 2. Representative transthoracic echocardiographic images after transapical beating-heart septal myectomy. The echocardiographic images showing resting LVOTG and SAM at baseline (top) and 12 months (bottom) after TA-BSM. A4C color, color Doppler under the apical 4-chamber view.
